# Novel fungal species associated with *Arundo donax* (Poales, Poaceae) in Jiangxi, China

**DOI:** 10.3897/mycokeys.136.194074

**Published:** 2026-07-07

**Authors:** YinRu Xiong, Ishara S. Manawasinghe, MiaoMiao Zhang, Qiong Ren, LiYing Zhou

**Affiliations:** 1 Wetland Ecological Resources Research Center, Jiangxi Academy of Forestry, National Ecosystem Research Station of Jiangxi Wugong Mountain Meadow, Jiangxi, Nanchang 330013, China Center of Excellence in Fungal Research, Mae Fah Luang University Chiang Rai Thailand; 2 Center of Excellence in Fungal Research, Mae Fah Luang University, Chiang Rai 57100, Thailand Beijing Key Laboratory of Environment-Friendly Management on Fruit Diseases and Pests in North China, Institute of Plant Protection, Beijing Academy of Agriculture and Forestry Sciences Beijing China; 3 Beijing Key Laboratory of Environment-Friendly Management on Fruit Diseases and Pests in North China, Institute of Plant Protection, Beijing Academy of Agriculture and Forestry Sciences, Beijing 100097, China Wetland Ecological Resources Research Center, Jiangxi Academy of Forestry, National Ecosystem Research Station of Jiangxi Wugong Mountain Meadow Nanchang China

**Keywords:** Asexual morph, phylogeny, saprobic fungi, taxonomy, two new species

## Abstract

*Arundo donax* is a gramineous plant with economic value and green energy potential. However, the fungal resources of *A. donax* have rarely been explored. In 2025, fungi on *A. donax* were collected and isolated from wetlands in Jiangxi Province, China. Two saprobic fungal isolates were identified based on morphological characteristics and multigene phylogeny, combined with DNA sequence data of the internal transcribed spacer (ITS), part of the large subunit nuclear rRNA gene (LSU), the translation elongation factor 1-alpha gene (*tef*1-α), and the second largest subunit of the RNA polymerase II gene (*rpb*2). Two new species, *Lentimurispora arundinis* and *Stachylidium arundinis*, are reported. Illustrations, detailed descriptions, and in-depth phylogenetic analyses of all identified species are provided.

## Introduction

*Arundo donax* is a perennial herbaceous plant that is mainly found in tropical and subtropical regions (Yan et al. 2025). As a plant with extremely strong adaptability, capable of tolerating poor soil, low temperatures, and saline-alkali environments (Fatima et al. 2025), it has extremely high comprehensive utilization value and can be widely applied in ecological management and production promotion (Yan et al. 2025). In addition, *Arundo donax* also provides an excellent substrate for a variety of fungi, especially edible and medicinal fungi (Sidana et al. 2024; Wang et al. 2025). Nevertheless, published literature and empirical records concerning the biodiversity of saprobic microfungi remain relatively sparse (Baker et al. 2002; Hosoya and Tanaka 2007; Tibpromma et al. 2017), with both *Lentimurispora* and *Stachylidium* lacking host records on *Arundo donax*.

*Stachylidium* was initially proposed by Link (1809) but lacked specified details, resulting in the genus undergoing extensive study and revision. Fries (1832) subsequently designated *S. bicolor* as the type species, a taxonomic recommendation that has been upheld by numerous subsequent researchers (Dewi 2006; Giraldo and Crous 2019). Species of *Stachylidium* are primarily found on herbaceous and woody substrates, with occasional reports from soil environments (Hughes 1951; Barron 1968). To date, only the asexual morph has been documented, characterized by septate, verticillate conidiophores with a pale brown to brown base and a paler to hyaline, roughened apex; hyaline or pale brown, cylindrical to ellipsoid conidiogenous cells arranged in whorls; and cylindrical, unilocular, pale brown to brown conidia (Hughes 1951; Dewi 2006; Giraldo and Crous 2019). Although recent taxonomic compilations register seven species within the genus (Hyde et al. 2024), clear consensus has historically been established only for the morphological distinctions separating *S. bicolor* and *S. pallidum* (Kirk et al. 2008; Seifert and Gams 2011; Whitton et al. 2012). The remaining morphological species require further revision to ascertain their accurate taxonomic status (Kirk et al. 2008; Seifert and Gams 2011).

The monotypic genus *Lentimurispora* was introduced by Liu et al. (2018) and typified with *L. urniformis*, characterized by micronematous conidiophores, monoblastic conidiogenous cells, and muriform, lenticular conidia with dark brown central cells and paler peripheral cells, without known sexual morphs. *Lentimurispora* resembles *Hermatomyces* in having lenticular, muriform conidia, with subhyaline to pale brown peripheral cells and dark brown central cells (Ellis 1971; Tibpromma et al. 2016). However, *Lentimurispora* has micronematous conidiophores and hyaline, wedge-shaped conidiogenous cells, while *Hermatomyces* has short, pale brown conidiophores and cylindrical conidiogenous cells (Ellis 1971; Liu et al. 2018).

In this study, samples of *Lentimurispora* and *Stachylidium* were isolated from rotting *Arundo donax* collected from a wetland area in Jiangxi Province, China. Morphological studies and phylogenetic analyses of multiple gene loci confirmed that they were distinct from other known species. This study represents the first report of *Lentimurispora* and *Stachylidium* on *Arundo donax* and the first report from Jiangxi Province, China.

## Materials and methods

### Isolation and morphological characterization

During a survey of saprobic fungi on *Arundo donax* in Jiangxi Province, China, in 2025, samples were brought into the laboratory using plastic ziplock bags. Macro- and micro-characteristics were photographed using a ZEISS SteREO Discovery V20 stereomicroscope (Germany), a Nikon Eclipse 80i microscope, and the industrial Digital Sight DS-Fi1 (Panasonic, Japan) microscope. Following the methods of Senanayake et al. (2018, 2020), single-spore isolation was performed. Germinated spores were aseptically transferred onto fresh potato dextrose agar (PDA) plates and incubated at 25 °C to obtain pure cultures (Senanayake et al. 2020). The cultures obtained during the study were deposited in the culture collection of Zhongkai University of Agriculture and Engineering (**ZHKUCC**). Herbarium materials were deposited at the Mycological Herbarium of Zhongkai University of Agriculture and Engineering (**MHZU**). Faces of Fungi (**FoF**) numbers and Index Fungorum (**IF**) numbers were obtained as explained in Jayasiri et al. (2015) and Index Fungorum (2026).

Digital images of micromorphological structures, including shape, size, and color, were recorded. Measurements of structures, including spore dimensions for each species, were conducted using NIS-Elements BR 5.30.03. Adobe Photoshop CC 2019 and Adobe Illustrator CC 2019 software (Adobe Systems Inc., San Jose, USA) were used to develop images and make photo plates. All pure cultures obtained in this study were grown on potato dextrose agar (PDA) at 25 °C under 12 h of daylight for a week, and the diameter of the culture was measured after six weeks. AxioVersion Rel. 4.8 was used to take photos of the cultures.

### DNA extraction, PCR amplification, and sequencing

Pure cultures were grown on PDA plates for 1–2 weeks, and approximately 500 mg of fresh fungal mycelia were scraped. Total genomic DNA was extracted from the mycelia using the MagPure Plant AS Kit (Magen Biotech, China) following the manufacturer’s instructions. The primer sets LR0R/LR5 (Vilgalys and Hester 1990) were used to amplify the large subunit of the nuclear ribosomal RNA genes (LSU), NS1/NS4 (White et al. 1990) for the small subunit of the nuclear ribosomal RNA (SSU), ITS5/ITS4 (White et al. 1990) for the internal transcribed spacer region (ITS), EF1-728F/EF1-986R (Carbone and Kohn 1999) for translation elongation factor 1-alpha (*tef*1-α), and fRPB2-5F/fRPB2-7cR (Liu et al. 1999) for the second largest subunit of RNA polymerase II (*rpb*2), using the primers shown in Table 1.

**Table 1.** Gene regions and primer pairs used in this study.

**Locus**	**Primer Reaction condition (5’-3’)**	**References**
ITS	ITS5: 5’-DDAAGTAAAAGTCGTAACAAGG-3’ (Forward)	White et al. 1990
	ITS4: 5’-TCCTCCGCTTATTGATATGC-3’(Reverse)	
LSU	LR0R: 5’-ACCCGCTGAACTTAAGC-3’ (Forward)	Vilgalys and Hester. 1990
	LR5: 5’-TCCTGAGGGAAACTTCG-3’ (Reverse)	
*tef* 1-α	EF1-728F: 5’-CATCGAGAAGTTCGAGAAGG-3’ (Forward)	Carbone and Kohn 1999
	EF1-986R: 5’-TACTTGAAGGAACCCTTACC-3’ (Reverse)	
*rpb*2	fRPB2-5F: 5’-GAYGAYMGWGATCAYTTYGG-3’ (Forward)	Liu et al. 1999
	RPB2-7cr: 5’-CCCATRGCTTGYTTRCCCAT-3’ (Reverse)	

The PCR reaction mixture contained a total volume of 25 μL, which consisted of 12.5 μL of 2× FastTaq Premix (mixture of FastTaq™ DNA Polymerase, buffer, dNTP mixture, and stabilizer) (Beijing Qingke Biological Technology Co., Ltd., Beijing, PR China), 1 μL each of forward and reverse primers, 9.5 μL of ddH_2_O, and 1 μL of DNA. Polymerase chain reaction (PCR) was performed in a C1000 Touch™ thermal cycler. The PCR procedure was as follows: for ITS/LSU/SSU, the initial denaturation step was performed at 95 °C for 2 min, followed by 35 amplification cycles at 95 °C for 1 min, 50 °C for 1 min, and 72 °C for 1 min, with a final extension at 72 °C for 10 min. For *tef*1-α/*rpb*2, an initial step of 2 min at 95 °C was followed by 35 cycles of 1 min at 95 °C, 1 min at 52 °C, and 1 min at 72 °C, with a final extension at 72 °C for 7 min. After PCR amplification, the product was observed on a 1% agarose gel under ultraviolet light. DNA sequencing was completed by Tianyi (Guangzhou, China) Co., Ltd. New sequences were deposited in GenBank. The sequences used for analyses, with accession numbers, are given in Table 2.

**Table 2.** Taxon names, strain numbers, and corresponding GenBank accession numbers of the taxa used in the phylogenetic analyses.

**Taxon**	**strain**	**GenBank accession number**			
		**ITS**	**LSU**	***tef* 1-α**	***rpb*2**
***Acremoniisimulans cocois***	**MFLU 15-2350**	**ON650683**	**NG_228973**	**NA**	**NA**
***Acremoniisimulans hongheensis***	**HKAS 122669**	**OQ379005**	**NG_242065**	**OQ378995**	**OQ378988**
***Acremoniisimulans thailandensis***	**MFLU 18-0012**	**NR_185553**	**NG_228789**	**NA**	**NA**
***Allomusicillium domschii***	**CBS 764.69**	**NR_189434**	**NG_242038**	**OQ470787**	**OQ453889**
*Angustimassarina kunmingense*	KUNCC 22-10799	ON352672	ON352671	ON364144	NA
*Bahusandhika indica*	MFLU 23-0494	PP657268	PP657303	PP817245	NA
***Brunneochlamydosporium cibotii***	**CBS 109240**	**LR026678**	**LR025807**	**LR026380**	**NA**
***Brunneochlamydosporium macroclavatum***	**CBS 101249**	**LR026682**	**LR025811**	**LR026384**	**NA**
*Brunneoclavispora camporesii*	MFLUCC 11-0001	MN809329	MN809328	NA	NA
***Brunneomyces polyphialidus***	**CBS 166.80**	**NR_189435**	**OQ055413**	**OQ470791**	**OQ453893**
***Brunneomyces pseudozeylanicus***	**CBS 560.73**	**NR_189436**	**NG_056989**	**OQ470792**	**OQ453894**
*Flabellascoma fusiforme*	MFLUCC 18-1584	MN304830	MN274567	MN328902	NA
***Fuscohypha expansa***	**CBS 418.89**	**LR026715**	**LR025845**	**LR026412**	**LR026136**
***Fusiformiseptata crocea***	**MFLUCC 18-1415**	**MT627694**	**MN913722**	**MT954367**	**NA**
***Lectera capsici***	**CBS 142534**	**NR_155338**	**NG_058474**	**LR026454**	**LR026166**
***Lectera longa***	**IMI 181698**	**NR_111715**	**NG_066392**	**LR026458**	**NA**
***Lectera nordwiniana***	**CBS 144921**	**NR_161150**	**NG_066300**	**MK047570**	**MK047549**
***Lentimurispora arundinis***	**KUNCC 25-21579**	**PZ522736**	**PZ518409**	**PZ517818**	**PZ517822**
*Lentimurispora arundinis*	KUNCC 25-21580	PZ522737	PZ518410	PZ517819	PZ517823
***Lentimurispora urniformis***	**MFLUCC 18-0497**	**NA**	**MH179144**	**MH188055**	**NA**
*Magnibotryascoma kunmingense*	HKAS 111919	MW424770	MW424785	MW430106	MW430113
***Musicillium elettariae***	**CBS 252.80**	**OR922577**	**OR922581**	**NA**	**NA**
***Musicillium theobromae***	**CBS 968.72**	**MH860633**	**MH872329**	**LR026468**	**LR026178**
*Neomassarina pandanicola*	MFLUCC 16-0270	MG298945	MG298946	NA	NA
*Neomassarina thailandica*	MFLUCC 10-0552	KX672152	KX672157	KX672163	NA
*Neoroussoella fulvicomae*	MFLUCC 17-2073	MT310633	MT214588	MT394646	MT394702
*Neothyrostroma encephalarti*	CPC 35999	MN562104	MN567612	MN556831	NA
***Paragibellulopsis chrysanthemi***	**MAFF 242621**	**NR_155113**	**NG_067526**	**KC287232**	**NA**
***Parateichospora phoenicicola***	**CBS 147084**	**MZ064455**	**MZ064512**	**NA**	**MZ078212**
***Paucispora xishanensis***	**HKAS 115905**	**MZ966267**	**OK017522**	**MZ997340**	**NA**
*Paucispora xishanensis*	HKAS 115906	MZ966268	OK017523	MZ997341	NA
***Phaeoseptum hydei***	**MFLUCC 17-0801**	**MT240622**	**MT240623**	**MT241506**	**NA**
*Phaeoseptum mali*	KUMCC 21-0335	OL413027	OL413028	OL690512	NA
***Plectosphaerella citrullae***	**CBS 131741**	**LR026796**	**LR025934**	**LR026491**	**LR026197**
***Plectosphaerella cucumerina***	**CBS 137.33**	**LR026797**	**LR025935**	**LR026492**	**LR026198**
*Pleopunctum ellipsoideum*	MFLUCC 19-0390	MK804512	MK804517	MK828510	NA
***Pleopunctum menglaense***	**KUMCC 21-0026**	**ON009119**	**ON009103**	**NA**	**NA**
***Profundisphaeria fusiformispora***	**GZAAS 20-4010**	**NA**	**OP099534**	**OR140432**	**OR146942**
*Profundisphaeria fusiformispora*	GZAAS 20-4012	NA	OR209667	OR140433	NA
*Pseudomassarina clematidis*	MFLU 16-0493	MT415397	MT214586	MT394644	MT394700
***Pseudopyrenochaeta terrestris***	**CBS 282.72**	**NR_160575**	**NG_063947**	**NA**	**LT623287**
*Pseudoroussoella chromolaenae*	MFLUCC 17-1492	MT214345	MT214439	MT235769	NA
***Sodiomyces magadiensis***	**CBS 137619**	**NR_155791**	**NG_059967**	**NA**	**NA**
***Sodiomyces tronii***	**CBS 137618**	**NR_155790**	**KJ443147**	**NA**	**NA**
*Sparticola irregularis*	GZCC 23-0593	OR098709	OR091330	OR494044	OR494047
*Sparticola muriformis*	MFLUCC 17-0316	KY768864	KY768862	KY768874	KY855380
***Stachylidium arundinis***	**KUNCC 25-21577**	**PZ522734**	**PZ518407**	**PZ517816**	**PZ517820**
*Stachylidium arundinis*	KUNCC 25-21578	PZ522735	PZ518408	PZ517817	PZ517821
***Stachylidium bicolor***	**CBS 121802**	**LR026834**	**LR025972**	**LR026532**	**NA**
***Stachylidium chayuense***	**HKAS 134942**	**PQ323341**	**PQ323343**	**PQ563351**	**PQ563349**
***Stachylidium chayuense***	**HKAS 134941**	**PQ323342**	**PQ323344**	**PQ563352**	**PQ563350**
*Stachylidium pallidum*	DAOM 226658	LR026838	GU180651	LR026534	LR026228
*Teichospora thailandica*	MFLUCC 13-0284	KP899141	KP888647	KR075167	NA
***Trichosphaeria pilosa***	**CPC42927**	**OQ990134**	**OQ990085**	**OQ989249**	**OQ989224**
***Verticillium bjoernoeyanum***	**CBS149167**	**ON603786**	**ON603806**	**ON605634**	**ON605626**
***Verticillium isaacii***	**CBS130343**	**LR026899**	**NG_069485**	**LR026577**	**NA**
***Verticillium klebahnii***	**CBS130344**	**NR_126128**	**NG_069486**	**LR026578**	**NA**
***Xenoplectosphaerella clematidis***	**MFLU17-1475**	**NR_172181**	**NG_071256**	**MT394674**	**MT394722**

Ex-type strains are indicated in bold. Newly generated sequences are indicated with light red background. “NA” indicates that information is unavailable.

### Phylogenetic analyses

The DNA sequences were checked in Geneious Prime v. 2021.0.3 (Biomatters Ltd., San Diego, CA, USA) to ensure sequence quality and combine the sequences generated by forward and reverse primers. The BLASTn tool (Basic Local Alignment Search Tool) in the search engine of the National Center for Biotechnology Information (NCBI) was used to analyze the sequences obtained in this study (https://blast.ncbi.nlm.nih.gov/Blast.cgi). Based on NCBI BLAST results and previous publications, the sequences for the phylogenetic analysis were downloaded from GenBank and listed in Table 2. Phylogenetic analysis was conducted using Bayesian analysis in MrBayes v. 3.1.2 (Huelsenbeck and Ronquist 2001) and maximum likelihood (ML) based on the datasets, including reference DNA sequences and newly generated DNA sequences, using OFPT (Zeng et al. 2023) with the following protocol. Datasets of each gene region were first independently aligned with the “auto” strategy, based on data size, by MAFFT (Katoh and Standley 2013) and trimmed with the “gappyout” method, based on gap distribution, by TrimAl (Capella-Gutiérrez et al. 2009). The best-fit nucleotide substitution models for each dataset were then selected based on the Bayesian information criterion (BIC) from 22 common DNA substitution models with rate heterogeneity by ModelFinder (Kalyaanamoorthy et al. 2017). Afterward, all datasets were concatenated with partition information for subsequent phylogenetic analyses. Maximum likelihood with 1,000 replicates was performed using ultrafast bootstrap approximation (Hoang et al. 2018) with the SH-like approximate likelihood ratio test (SH-aLRT) (Guindon et al. 2010) by IQ-TREE (Nguyen et al. 2015). The consensus tree was summarized based on the extended majority rule. Bayesian inference was performed with two parallel Metropolis-coupled Markov chain Monte Carlo runs, each with one “cold” chain and three heated chains, by MrBayes (Ronquist et al. 2012), with trees sampled every 100 generations. The consensus tree was summarized after discarding the first 25% of samples when the average standard deviation of split frequencies fell below 0.01.

The best model of evolution was determined by MrModeltest v. 2.2 (Nylander 2008) for each gene. Maximum likelihood analyses were accomplished using RAxML-HPC2 on XSEDE v. 8.2.8 (Stamatakis et al. 2008; Stamatakis 2014) on the CIPRES Science Gateway platform (Miller et al. 2010) using the GTR+I+G model of evolution with 1,000 non-parametric bootstrapping iterations. MrBayes v. 3.0b4 was used for the Bayesian analyses (Huelsenbeck and Ronquist 2001). The Bayesian Markov chain Monte Carlo sampling (BMCMC) analysis was conducted with four simultaneous Markov chains. The best model of evolution was determined for each gene region of each phylogenetic analysis tree by MrModelTest v. 2.2 (Nylander 2008). For each phylogenetic analysis tree, the analysis was run for 1,000,000 generations, sampling the trees every 100^th^ generation. From the 10,000 trees obtained, the first 2,000, representing the burn-in phase, were discarded. The remaining 8,000 trees were used for calculating posterior probabilities in the majority-rule consensus tree. Taxonomic species were submitted to the Faces of Fungi database (Jayasiri et al. 2015).

## Results

### Taxonomy and phylogeny

**Trichosphaeriales M.E. Barr, Mycologia 75: 11 (1983)**

#### Trichosphaeriaceae G. Winter, Rabenhorst’s Kryptogamen-Flora, Pilze - Ascomyceten Ed. 2, 1 (1): 191 (1884)

***Stachylidium* Link, Mag. Neuesten Entdeck. Gesammten Naturk. Ges. Naturf. Freunde Berlin 3 (1): 15 (1809)**

##### Stachylidium arundinis

Taxon classificationFungiGlomerellalesTrichosphaeriaceae

Y.R. Xiong, L.Y. Zhou & M.M. Zhang sp. nov.

F1E1066A-3F3E-5DFE-B12B-A7BB6CB50C5E

Index Fungorum: IF905641

Facesoffungi Number: FoF20125

Fig. 2

###### Etymology.

Species epithet refers to the host genus name “*Arundo*” from which the holotype was isolated.

###### Description.

Saprobic on dead petiole of *Arundo donax*. **Asexual morph**: Hyphomycetous. Colonies on the substrate, brown, white at middle and apex, irregular, hair, dry. ***Conidiophores*** 140–290 × 5–6 μm (x̄ = 198 × 5.2 μm, *n* = 25), simple, erect, unbranched, brown at the base, paler to hyaline toward the apex, with lateral branches arising in the upper part. ***Branches*** cylindrical, taper gradually to the apex, pale brown to hyaline. ***Lateral branches*** bearing singly, in pairs or in verticils of 2 to 3 secondary branches or phialides. ***Conidiogenous cell*** 10–12 × 3–3.5 μm (x̄ = 11.3 × 3.2 μm, *n* = 25), holoblastic, cylindrical, constricted at base, conoidal at the apex, hyaline to subhyaline, smooth. ***Conidia*** 4.5–6 × 2–2.5 μm (x̄ = 5.6 × 2.2 μm, *n* = 25), hyaline, cylindrical, granulate, smooth and thin-walled. **Sexual morph**: Undetermined.

###### Culture characteristics.

Colonies on PDA fast growing, after two weeks reaching 4–5 cm diam. at 25 °C, white at the edge, cream in the middle and outwardly strongly radiating, irregular, radially striated with lobate edge.

###### Material examined.

China • Jiangxi Province, Jingdezhen City, on dead petiole of *Arundo donax*, 9 Sep 2025, Y.R. Xiong and L.Y. Zhou, XG503 (HKAS 155056, holotype); ex-type, KUNCC 25-21577, other living culture KUNCC 25-21578.

###### Notes.

**Figure 1.**
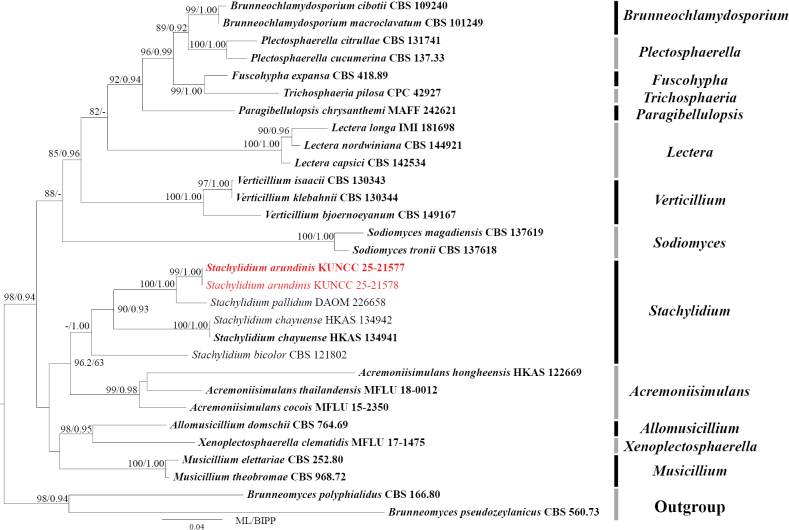
Phylogram generated from maximum likelihood analysis of Trichosphaeriaceae based on the combined ITS, LSU, *rpb*2, and *tef*1-α sequence dataset, with two *Brunneomyces* taxa as the outgroup. Bootstrap support for maximum likelihood (ML) equal to or greater than 75% and Bayesian inference posterior probability (BIPP) equal to or greater than 0.90 are indicated above the branches as ML/BIPP. The scale bar indicates 0.04 nucleotide changes per site. Isolates from this study are marked in red, and ex-type strains are indicated in bold.

Two isolates from this study formed a separate lineage and clustered with *Stachylidium pallidum* (DAOM 226658) in the phylogenetic tree with 100% ML and 1.00 BIPP support (Fig. 1). The nucleotide differences excluding gaps between *S. arundinis* (KUNCC 25-21577) and *S. pallidum* (DAOM 226658) were as follows: ITS = 0.06% (3/488 bp), LSU = 0.11% (1/880 bp), *tef*1-α = 2.92% (23/787 bp), and *rpb*2 = 7.94% (59/743 bp), with the remaining nucleotide positions identical. Morphologically, *S. arundinis* is characterized by lateral branches arising in the upper part, which differs from *S. pallidum*, which has strongly branched conidiophores and an apex with an almost imperceptible minute collarette (Dewi 2006; Giraldo and Crous 2019). Based on the phylogenetic placement and morphological variations, *S. arundinis* is introduced as a new species.

**Figure 2.**
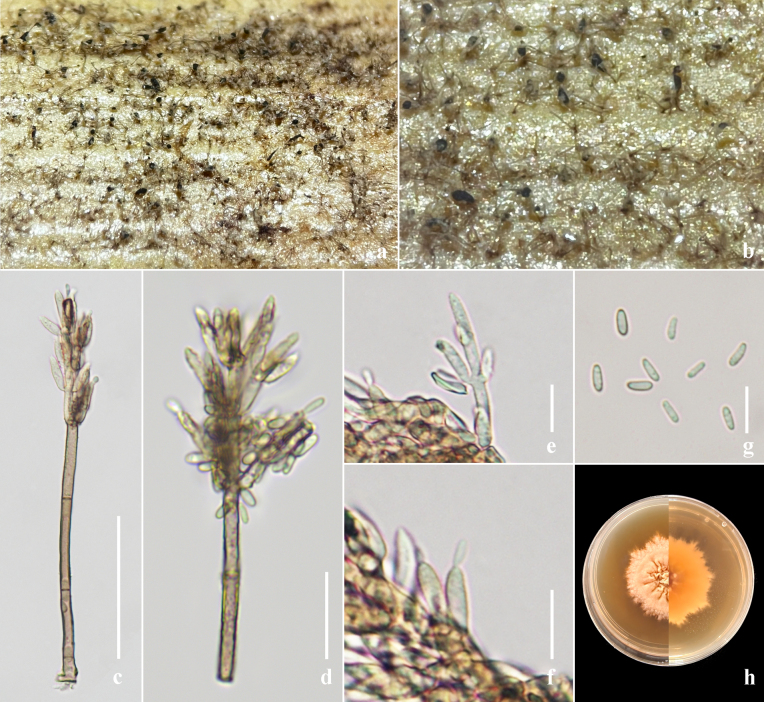
*Stachylidium arundinis* (holotype, HKAS 155056). **a, b**. Appearance of sporodochia on host substrate. **c, d**. Conidiophores and conidiogenous cells with conidia. **e, f**. Conidiogenous cells and developing conidia. **g**. Conidia. **h**. Culture on PDA from above and reverse. Scale bars: 50 μm (**c**); 20 μm (**d**); 10 μm (**e–g**).

#### Pleosporales Luttr. ex M.E. Barr, Prodromus to class Loculoascomycetes: 67 (1987)

**Lentimurisporaceae N.G. Liu, J.K Liu & K.D. Hyde, Cryptog. Mycol. 39 (2): 270 (2018)**

***Lentimurispora* N.G. Liu, Bhat & K.D. Hyde, Cryptog. Mycol. 39 (2): 270 (2018)**

##### Lentimurispora arundinis

Taxon classificationFungiPleosporalesLentimurisporaceae

Y.R. Xiong, L.Y. Zhou & M.M. Zhang sp. nov.

668B758E-400F-5C76-BC45-23C05EC97ED3

Index Fungorum: IF905642

Facesoffungi Number: FoF20126

Fig. 4

###### Etymology.

Species epithet refers to the host genus name “*Arundo*” from which the holotype was isolated.

###### Description.

Saprobic on dead petiole of *Arundo donax*. **Asexual morph**: Hyphomycetous. Colonies on substrate, superficial, black, scattered, gregarious, punctiform. Mycelium immersed in the substratum, composed of branched, septate, hyaline hyphae. ***Conidiophores*** micronematous, hyaline, most reduced to conidiogenous cells. ***Conidiogenous cells*** 17–38 × 7–12 μm (x̄ = 22.3 × 9.6 μm, *n* = 20), integrated, terminal, hyaline, smooth, wedge-shaped, clavate, truncate at apex after conidial secession. ***Conidia*** 11–15 × 17–21 μm (x̄ = 13.2 × 19.2 μm, *n* = 30), acrogenous, thick-walled, smooth, multiseptate, cushion-like, muriform, slightly constricted at the septa, median with black ellipsoid area, most 3-layer subhyaline or pale brown peripheral cells; with a big attachment cell, hyaline, thick-walled, inverted vase-like, clavate, narrowed towards the base, truncate at apex. **Sexual morph**: Undetermined.

###### Culture characteristics.

Colonies on PDA fast growing, after two weeks reaching 3–4 cm diam. at 25 °C, white at the edge, gray in the middle, entire edge, curled, umbonate.

###### Known distribution.

China.

###### Material examined.

China • Jiangxi Province, Jiujiang City, on dead petiole of *Arundo donax*, 14 Nov 2025, Y.R. Xiong and L.Y. Zhou, XG518 (HKAS 155057, holotype); ex-type, KUNCC 25-21579, other living culture KUNCC 25-21580.

###### Notes.

**Figure 3.**
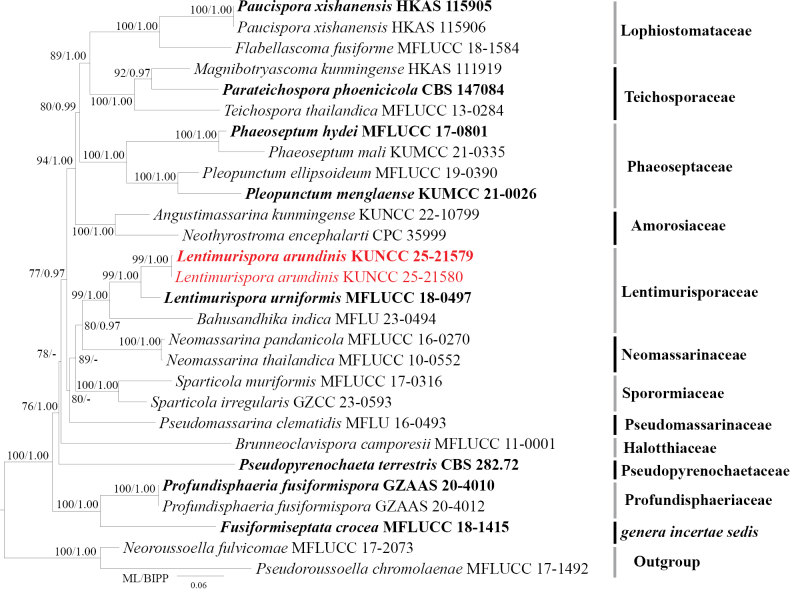
Phylogram generated from maximum likelihood analysis of selected Pleosporales based on the combined ITS, LSU, *rpb*2, and *tef*1-α sequence dataset, with one *Neoroussoella* taxon and one *Pseudoroussoella* taxon as the outgroup. Bootstrap support for maximum likelihood (ML) equal to or greater than 75% and Bayesian inference posterior probability (BIPP) equal to or greater than 0.90 are indicated above the branches as ML/BIPP. The scale bar indicates 0.04 nucleotide changes per site. Isolates from this study are marked in red, and ex-type strains are indicated in bold.

Two isolates from this study formed a separate lineage and clustered with *Lentimurispora urniformis* (MFLUCC 18-0497) in the phylogenetic tree with 99% ML and 1.00 BIPP support (Fig. 3). The nucleotide differences excluding gaps between *L. arundinis* (KUNCC 25-21579) and *L. urniformis* (MFLUCC 18-0497) were as follows: LSU = 1.54% (14/911 bp), SSU = 0.49% (5/1027 bp), and *tef*1-α = 5.47% (50/914 bp). Morphologically, *Lentimurispora arundinis* is characterized by conidia with a black ellipsoid area in the median region and mostly three-layered subhyaline or pale brown peripheral cells. However, *L. urniformis* differs from *L. arundinis* by its lenticular conidia with dark brown central cells and subhyaline to pale brown peripheral cells (Liu et al. 2018). Based on the phylogenetic placement and morphological variations, *L. arundinis* is introduced as a new species.

**Figure 4.**
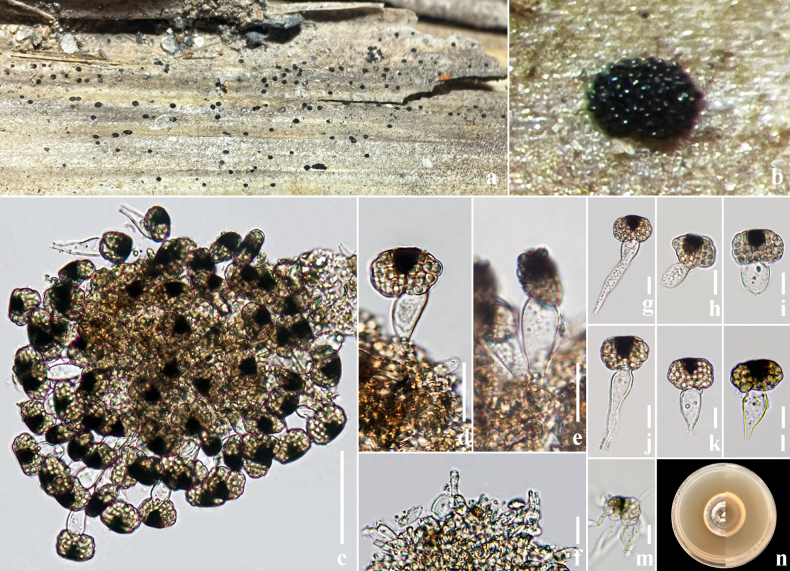
*Lentimurispora arundinis* (holotype, HKAS 155057). **a, b**. Appearance of sporodochia on host substrate. **c–f**. Conidiogenous cells and developing conidia. **g–l**. Conidiogenous cells and developing conidia. **m**. Germinated conidium. **n**. Culture on PDA from above and reverse. Scale bars: 50 μm (**c**); 20 μm (**d, e**); 10 μm (**f–m**).

## Discussion

In the present study, two novel fungal species, *Lentimurispora arundinis* and *Stachylidium arundinis*, are introduced based on specimens collected from *Arundo donax* in China. Although numerous fungal species have been recorded on *A. donax*, only two, *Bipolaris sorokiniana* and *Corynespora donacis*, have been accepted in China (Kumar et al. 2021; Wang and Wei 2016). Despite an extensive historical record of fungi associated with *A. donax* worldwide, the discovery of novel species and new host affiliations has remained remarkably limited over the past decade (Mehrabi et al. 2017; Tibpromma et al. 2017; Moyo et al. 2018a, 2018b; Lombard et al. 2019; Crous et al. 2020; Hao et al. 2020; Kumar et al. 2021). Considering that plant-host affinity and regional microclimates are generally recognized as important factors influencing fungal diversity (Hawksworth and Lücking 2017), it is reasonable to infer that a considerable number of undiscovered fungal taxa associated with *A. donax* may still exist, particularly within subtropical and temperate ecosystems.

In addition to species-level novelties, the morphological re-evaluations in this study also prompt a reconsideration of generic boundaries. A comprehensive comparison with allied taxa indicates that the generic circumscription of *Lentimurispora* provided by Liu et al. (2018) may warrant further refinement. It appears that the genus could be more accurately characterized by the presence of a prominent basal attachment cell on the conidia, rather than the previously emphasized wedge-shaped conidiogenous cells. Morphologically, this large, hyaline, appendage-like structure is hypothesized to function as an adhesion mechanism, which likely facilitates the adherence of *Lentimurispora* conidia to various substrate surfaces (Braun and Howard 1994; Osherov and May 2001; Yang et al. 2017; Goh et al. 2024). Such morphological features are frequently observed in saprobic microfungi as adaptive strategies for colonization in specific microhabitats (Jones 2006).

At the generic level, the discovery of *S. arundinis* further highlights the taxonomic complexities within *Stachylidium*. Established in the 19^th^ century, *Stachylidium* has historically posed challenges for species delimitation, primarily due to interspecific morphological similarities and the lack of ex-type molecular data for several older epithets (Hughes 1951; Giraldo and Crous 2019). In this context, *S. arundinis* demonstrates both considerable molecular divergence from its phylogenetically close relative, *S. pallidum*, and distinct morphological traits, notably by producing secondary conidiogenous cells exclusively at the apex of the conidiophore (Giraldo and Crous 2019; Ma et al. 2025). This finding corroborates that the integration of multilocus phylogeny with detailed morphological observation remains an effective approach for resolving cryptic species complexes within historically ambiguous genera (Jeewon and Hyde 2016).

Geographically, this study represents the first report of microfungi associated with *A. donax* in Jiangxi Province, thereby expanding the known distribution of this host-mycobiota association. *Arundo donax* is increasingly valued for its ecological and industrial applications; it contributes to carbon sequestration, enhances wetland carbon sinks, and shows potential as a biomass source for hydrogen production (Allesina et al. 2018; Vasmara et al. 2025). Given that plant-associated mycobiomes are known to be involved in nutrient cycling and biomass degradation (Hyde et al. 2019), continued investigation into the fungal diversity of *A. donax* is of significant importance for exploring microbial–plant interactions. Ultimately, such baseline taxonomic and ecological data may provide valuable scientific references for optimizing the management and industrial utilization of this plant resource.

## Supplementary Material

XML Treatment for Stachylidium arundinis

XML Treatment for Lentimurispora arundinis

## Additional information

### Artificial Intelligence (AI) use

The authors accept full responsibility for the content of the manuscript, including the disclosure of any use of AI.

No AI tools were used in the preparation of this manuscript.

### Author ORCIDs

Y.R. Xiong https://orcid.org/0000-0002-4673-606X

I.S. Manawasinghe https://orcid.org/0000-0001-5730-3596

### Data availability

All of the data that support the findings of this study are available in the main text.
